# HIV-1 resists MxB inhibition of viral Rev protein

**DOI:** 10.1080/22221751.2020.1818633

**Published:** 2020-09-20

**Authors:** Zhen Wang, Keli Chai, Qian Liu, Dong-Rong Yi, Qinghua Pan, Yu Huang, Juan Tan, Wentao Qiao, Fei Guo, Shan Cen, Chen Liang

**Affiliations:** a Lady Davis Institute, Jewish General Hospital, Montreal, Canada; b Department of Medicine, McGill University, Montreal, Canada; c Department of Microbiology and Immunology, McGill University, Montreal, Canada; d College of Life Sciences, Nankai University, Tianjin, People’s Republic of China; e Institute of Medicinal Biotechnology, Chinese Academy of Medical Science, Beijing, People’s Republic of China; f Institute of Pathogen Biology, Chinese Academy of Medical Science & Peking Union Medical College, Beijing, People’s Republic of China

**Keywords:** MxB, HIV-1, Rev, transportin 1, nuclear import, interferon

## Abstract

The interferon-inducible myxovirus resistance B (MxB) protein has been reported to inhibit HIV-1 and herpesviruses by blocking the nuclear import of viral DNA. Here, we report a new antiviral mechanism in which MxB restricts the nuclear import of HIV-1 regulatory protein Rev, and as a result, diminishes Rev-dependent expression of HIV-1 Gag protein. Specifically, MxB disrupts the interaction of Rev with the nuclear transport receptor, transportin 1 (TNPO1). Supporting this, the TNPO1-independent Rev variants become less restricted by MxB. In addition, HIV-1 can overcome this inhibition by MxB through increasing the expression of multiply spliced viral RNA and hence Rev protein. Therefore, MxB exerts its anti-HIV-1 function through interfering with the nuclear import of both viral DNA and viral Rev protein.

## Introduction

Myxovirus resistance (Mx) proteins were discovered for their protective function of mice from otherwise lethal infection by influenza viruses [1]. Humans have two Mx paralogs, MxA (also called Mx1) and MxB (or Mx2) [2]. While MxA is known to inhibit influenza viruses, thogotovirus, and vesicular stomatitis virus [3], MxB inhibits human immunodeficiency virus type 1 (HIV-1) [4–6], hepatitis C virus [7] and herpesviruses [8–10]. Their distinct antiviral spectra are largely attributed to an extended N-terminal sequence in MxB, since attaching this N-terminal sequence of MxB to MxA allows the MxA variant to inhibit HIV-1 [11]. Beyond this major difference in their sequences, as large GTPases of the dynamin-like superfamily, MxA and MxB share a structure consisting of a globular GTPase domain which is connected to a helical stalk domain via a flexible bundle-signaling element (BSE) [12–15]. Both the GTPase activity and protein multimerization through the stalk domain contribute to the antiviral functions of MxA and MxB [4,5,14,16–19].

The antiviral specificity of MxA and MxB is determined by their distinct ways of targeting viral proteins. MxA uses a region called loop 4 to interact with the nucleocapsid protein (NP) of influenza virus [20,21], whereas MxB binds to the capsid protein shell of HIV-1 through its N-terminal sequence together with the GTPase domain [14,15,22,23]. The above interactions occur within the cytoplasm, and impair the nuclear entry of viral genomes. In response, influenza viruses change the NP sequence to resist MxA inhibition [20,24,25]. Such MxA-resistant NP mutations have been identified in the avian influenza virus strains that have transmitted into humans [26,27]. Similarly, MxB-resistant mutations occur in the capsid protein (CA) of HIV-1 [4–6,28,29].

However, the molecular details remain elusive regarding how Mx proteins inhibit the nuclear entry of viral RNA or DNA. While certain HIV-1 CA mutants such as P90A are refractory to MxB inhibition, they are still efficiently bound by MxB [14,29]. It is postulated that HIV-1 changes the route of nuclear import by mutating the CA protein, thus avoids MxB restriction [30,31]. One implication of this theory is that MxB is more effective in deterring certain nuclear import pathways than others. However, elucidating the detailed mechanisms is hampered by the complexity of HIV-1 nuclear entry which is modulated by a large group of viral and cellular factors [32,33].

In addition to HIV-1 DNA, we conjectured that MxB may also affect the nuclear import of viral proteins. Indeed, in this study, we found that the nuclear import of HIV-1 Rev (regulator of expression of virion proteins) was impaired by MxB. With this unique and simple protein transport system, we were able to further show that MxB prevents the nuclear entry of HIV-1 Rev through blocking the interaction of Rev with transportin 1 (TNPO1).

## Materials and methods

### Plasmid DNA

The infectious HIV-1 DNA clone NL4-3 was obtained from the NIH AIDS Reagent program (catalog 114). The CA88 HIV-1 DNA contains the A88T mutation within the capsid protein [6]. The GPV-RRE, GPV-CTEx4, and Rev-RFP DNA clones were previously described [34]. The MxB-RFP and MxB-GFP DNA clones were constructed by attaching the fluorescent protein sequences in frame to the C-terminus of MxB protein. Over-lapping PCRs were performed to amplify MxB and RFP DNA using primer pairs 5’-ACT AAG ACC GGT ATG TCT AAG GCC CAC AAG CCT TG-3’ and 5’-TAA TCA GCT CTT CGC CCT TAG ACA CAG CGG CCG CGT GGA TCT CTT TGC TGG AGA ATT-3’, 5’-AAT TCT CCA GCA AAG AGA TCC ACG CGG CCG CTG TGT CTA AGG GCG AAG AGC TGA TTA-3’ and 5’-CTG TCC GGA TCC TCA ATT AAG TTT GTG CCC CAG-3’. The final PCR product was cloned into the pQCXIP vector (Clontech, catalog 631516). The MxB DNA was inserted into the pEGFP-N1 vector to generate MxB-GFP fusion protein. Rev and MxB mutants were engineered using the site-directed PCR mutagenesis method. The GFP-Vpr plasmid DNA was obtained from the NIH AIDS Reagent program (catalog 11386). The GFP-IN plasmid was generated by cloning the IN sequence from HIV-1 NL4-3 in frame to the 3’ end of the GFP gene in the pEGFP-C1 vector. Tat-GFP was created by cloning Tat DNA into the pEGFP-N1 expression vector. The lentiCRISPR(v2) (catalog 52961) [35], pNLS-mCherry-LEXY (catalog 72655) [36], and pLEXY (catalog 72656) plasmid DNA clones were obtained from Addgene. TNPO1 sgRNAs, 5’-GAA CCC ACA AGA TCA TTG AG-3’ (sgTNPO1-1) and 5’-ATC ACA ACT ATA GCC TCC AA-3’ (sgTNPO1-2), were cloned into lentiCRISPR(v2) to create TNPO1 knockdown cell lines as we previously described [37]. M9 PY-NLS (NQSSNFGPMKGGNFGGRSSGPYGGGGQYFAKPRN- QGGY) DNA sequence was inserted into pLEXY vector for live cell imaging. The TNPO1 siRNA (ID s7933) and KPNB1 siRNA (ID s7918) were purchased from Ambion. Leptomycin B (LMB) was purchased from Sigma (catalog L2913).

### Cell culture and transfection

HEK293T and HeLa cells were cultured in Dulbecco’s Modified Eagle Medium (DMEM) (Invitrogen) supplemented with 10% fetal bovine serum (FBS) (Invitrogen), 50 U/ml penicillin, and 50 μg/ml streptomycin (Invitrogen). SupT1 and Jurkat cells were cultured in RPMI1640 media supplemented with 10% FBS, 2 mM L-glutamine, 50 U/ml penicillin, and 50 μg/ml streptomycin. The MxB-expressing SupT1 cell line was created as previously described [6]. Transfection of plasmid DNA was performed with polyethylenimine (PEI) (Polysciences Inc, catalog 23966) in HEK293T cells using 100 ng GPV-RRE DNA, 12.5 ng Rev-RFP DNA, 25 ng MxB DNA in 12-well plates, or with Lipofetamine 3000 (Invitrogen) in HeLa cells using 40 ng MxB and 70 ng Rev-RFP DNA in 12-well plates, in accordance with manufacturer’s instruction. Transfection of siRNA was conducted in HeLa cells with Lipofectamine 3000 (Invitrogen) using 20 pmol siRNA in 12-well plates. Jurkat cells (2 × 10^6^) were transfected with 2 μg GPV-RRE and 1 μg Rev, or 2 μg GPV-CTEx4, together with 5 μg MxB, using the NEON Electroporation Transfection System (Thermo Fisher Scientific).

### Immunofluorescence staining and confocal microscopy

The transfected HeLa or HEK293T cells were washed with phosphate buffered saline (PBS) and fixed with 4% paraformaldehyde for 10 min, permeabilized with 0.5% Triton X-100/PBS for 10 min, and then blocked with 5% FBS/PBS for 1 h. MxB-FLAG was detected by incubation with mouse anti-FLAG antibody (Sigma, catalog F1804-200UG, 1:1000) for 2 h, followed by incubation with Alexa Fluor 488 conjugated donkey anti-mouse secondary antibody (Invitrogen, catalog A21202, 1:500) for 1 h. TNPO1 was detected using mouse anti-TNPO1 antibody (Abcam, catalog ab10303, 1:1000) for 2 h, then with Alexa Fluor 647-goat anti-mouse secondary antibody (Invitrogen, catalog A21237, 1:500) for 1 h. KPNB1 was detected with mouse anti-KPNB1 antibody (Invitrogen, catalog MA3-070, 1:500) for 2 h and Alexa Fluor 647 conjugated goat anti-mouse secondary antibody (Invitrogen, catalog A21237,1:500) for 1 h. DNA was stained with DAPI (4=,6-diamidino-2-phenylindole). Images were recorded with a Quorum WaveFX Spinning Disk Confocal using a 63x oil objective.

### In situ proximity ligation assay (PLA)

In situ PLA was carried out using Duolink^™^ In Situ Red Starter Kit Mouse/Rabbit (Sigma Aldrich, catalog DUO92101) according to the manufacturer’s protocol. Briefly, 4 × 10^5^ HeLa cells in 20 mm glass bottom dishes were transfected with 500 ng pRev-HA and 100 ng MxB-GFP or Δ(1-25) MxB-GFP using Lipofectamine 2000 (Invitrogen). After 48 h, cells were fixed with 4% paraformaldehyde for 30 min and permeabilized with 0.2% Triton X-100/PBS, and then incubated with blocking solution for 1 h prior to incubation with both rabbit anti-HA (Abcam, catalog ab9110, 1:1000) and mouse anti-TNPO1 antibodies (Abcam, catalog ab10303, 1:1000) together for 1 h at 37°C. Then, cells were incubated for 1 h at 37°C with donkey anti-mouse and donkey anti-rabbit secondary antibodies that are conjugated with Duolink PLA minus and plus oligonucleotide probes, and further incubated with ligation solution for 30 min at 37°C. After washing cells with wash buffer A, the amplification solution was added for 100 min at 37°C to amplify the ligated PLA probes, then washed with wash buffer B. Samples were mounted with Duolink PLA mounting medium containing DAPI. Fluorescence signals were acquired with a PerkinElmer Ultra View VoX confocal microscope using a 100x objective.

### Fluorescent in situ RNA hybridization

This assay was performed with RNAscope Multiplex Fluorescent Reagent Kit v2 (Advanced Cell Diagnostics, catalog 323100). 5 × 10^4^ HeLa cells were seeded in 35 mm dishes and transfected with 500 ng Rev-HA, 1000 ng GPV-RRE and 100 ng MxB-FLAG or Δ(1-25) MxB-FLAG using Lipofectamine 2000 (Invitrogen). Forty-eight hours after transfection, cells were fixed with 4% paraformaldehyde for 20 min, followed by RNAscope protease treatment for 10 min at room temperature. RNA was detected using RNAscope multiplex fluorescent v2 assay with Gag-RRE probes (Advanced Cell Diagnostics, catalog 476121-C3) according to the manufacture’s instruction. Then, Rev-HA and MxB-FLAG proteins were stained with mouse anti-HA (Santa Cruz Biotechnology, catalog7392, 1:100) or goat anti-FLAG (Abcam, catalog 1257, 1:1000) primary antibodies, and corresponding Alexa Fluor-555 conjugated donkey anti-mouse (Invitrogen, catalog A31570, 1:1000) or Alexa Fluor-647 conjugated donkey anti-goat (Invitrogen, catalog A21447, 1:1000) secondary antibodies. DNA was stained with DAPI. Images were acquired using a PerkinElmer Ultra View VoX confocal microscope using a 100x objective.

### Live cell imaging

HeLa cells were seeded into a 4-well 1.5H glass-bottom chamber (Ibidi) at a density of 1.5 × 10^4^ cells per well, before being transfected with 40 ng pNLS-mCherry-LEXY and 25 ng pEGFP or 40 ng pMxB-EGFP plasmid DNA. Cells were maintained at 37°C, 45% humidity and 5% CO_2_ in a Pathology Devices LIVECELL incubator (Pathology Devices, Inc.). Live cell imaging was conducted 20 h post transfection using a Nikon A1 confocal microscope. Before laser excitation, cells were kept in dark for 5 min, and then scanned with a 458 nm laser (∼2 µW intensity) for 30 ms every 10 s, which lasted 10 min, followed by dark recovery. mCherry was detected in every 30 s using the 561 nm laser for excitation. All images and movies were processed and analysed using NIS-Elements, Volocity and FIJI/NIH ImageJ2 softwares.

### Viral reverse transcriptase assay

Ten microliters of viral supernatants were incubated in a total 50 μl reaction mix containing 50 mM Tris-HCl (pH 7.9), 150 mM KCl, 5 mM MgCl_2_, 0.5 mM EGTA, 0.05% Triton X-100, 5 mM DTT, 0.3 mM GSH, 0.025 U poly(rA)/oligo(dT) (Midland Certified Reagent Company, catalog P-4012), and 5 μCi [^3^H] TTP (PerkinElmer, catalog NET221A005 MC). After 3 h incubation at 37°C, 10% TCA (150 μl) was added to precipitate the newly synthesized DNA at 4°C for 30 min, followed by passing through multiscreen glass fibre FC plate (Millipore) to collect the [^3^H]-labeled DNA. The level of incorporated [^3^H] was measured using the Beckman Scintillation Counter. The results reflect the amount of newly synthesized DNA by viral reverse transcriptase.

### HIV-1 infection of SupT 1 cells

HIV-1 NL4-3 and CA88 viruses were produced by transfecting the HEK293T cells. The amounts of viruses were determined by measuring viral reverse transcriptase activity. The same amounts of viruses were used to infect the control SupT1 cells or SupT1 cells expressing exogenous MxB. Forty hours after infection, cells were either lysed for protein extraction and Western blotting analysis, or dissolved in Trizol (Invitrogen, catalog 15596026) to extract RNA for RT-qPCR.

### Western blotting

The transfected HEK293T cells were harvested at 24 h post transfection, and infected SupT1 cells were harvested at 48 h post infection. Cells were lysed in RIPA buffer containing 50 mM Tris-HCl [pH 7.5], 150 mM NaCl, 1 mM EDTA, 1% Nonidet P-40, 0.1% SDS, and 1× protease inhibitor cocktail (Roche). An amount of 20 μg of cell lysates were resolved on 12% SDS-PAGE by electrophoresis. The separated proteins were transferred onto the PVDF membrane (Roche, catalog 3110040), followed by incubation with rabbit anti-HIV-1 p24/CA antibody (Sigma-Aldrich, catalog SAB3500946, 1:5000), mouse anti-FLAG antibody (Sigma Aldrich, catalog F1804-200UG, 1:5000), mouse anti-tubulin antibody (Santa Cruz Biotechnology, catalog sc-23948, 1:5000), mouse anti-GAPDH antibody (Santa Cruz Biotechnology, catalog sc-32233, 1:5000), rabbit anti-HIV-1 Rev antibody (AmericanBio, catalog 501(ABT), 1:5000), mouse anti-HIV-1 Tat antibody (NIH AIDS Reagents program, catalog 4672, 1:5000), mouse anti-HIV-1 Nef antibody (NIH AIDS Reagents program, catalog 1539, 1:5000). After washing with PBS, the membrane was further incubated with HRP-conjugated goat anti-mouse (SeraCare, catalog 5450-0011, 1:10000) or goat anti-rabbit (SeraCare, catalog 5450-0010, 1:10000) secondary antibodies. The protein signals were detected with enhanced chemiluminescence reagents (PerkinElmer) and exposure to X-ray films. The relative protein band intensities were quantified using the ImageJ Gel Analyser.

### RT-qPCR to determine levels of HIV-1 RNA

The detailed protocol was described by [38]. Briefly, total RNA from HIV-1 infected SupT1 cells was extract using Trizol. Reverse transcription of RNA was performed using the M-MLV (Moloney Murine Leukaemia Virus) reverse transcriptase (Invitrogen, catalog 28025013) and random hexamers (Invitrogen, catalog 8080127). Real-time PCR was performed with Fast SYBR Green Master mix (Invitrogen, catalog 4385612) on the 7500 Fast Real-Time PCR System (Applied Biosystems), using the following primer pairs as described in [39]: 5’-GAC GCT CTC GCA CCC ATC TC-3’ and 5’-CTG AAG CGC GCA CGG CAA-3’ to amplify unspliced full-length HIV-1 RNA, 5’- GGC GGC GAC TGG AAG AAG C-3’ and 5’- CTA TGA TTA CTA TGG ACC ACA C-3’ to amplify singly spliced HIV-1 RNA, 5’- GAC TCA TCA AGT TTC TCT ATC AAA-3’ and 5’- AGT CTC TCA AGC GGT GGT-3’ to amplify multiply spliced HIV-1 RNA, 5’-TTA GAC CAG ATC TGA GCC TGG GAG-3’ and 5’-GGG TCT GAG GGA TCT CTA GTT ACC-3’to amplify the TAR sequence, thus the total HIV-1 RNA, 5’- GAG CGG TTC CGC TGC CCT GAG GCA CTC-3’ and 5’- GGG CAG TGA TCT CCT TCT GCA TCC TG-3’ to amplify β-actin mRNA as the internal control.

### Statistical analysis

*P*-values were calculated with the Student t test. Pearson correlation coefficient (Pearson’s *r*) values of correlation analysis were calculated using the GraphPad Prism program.

## Results

### MxB causes cytoplasmic retention of HIV-1 Rev protein

**Figure 1.**
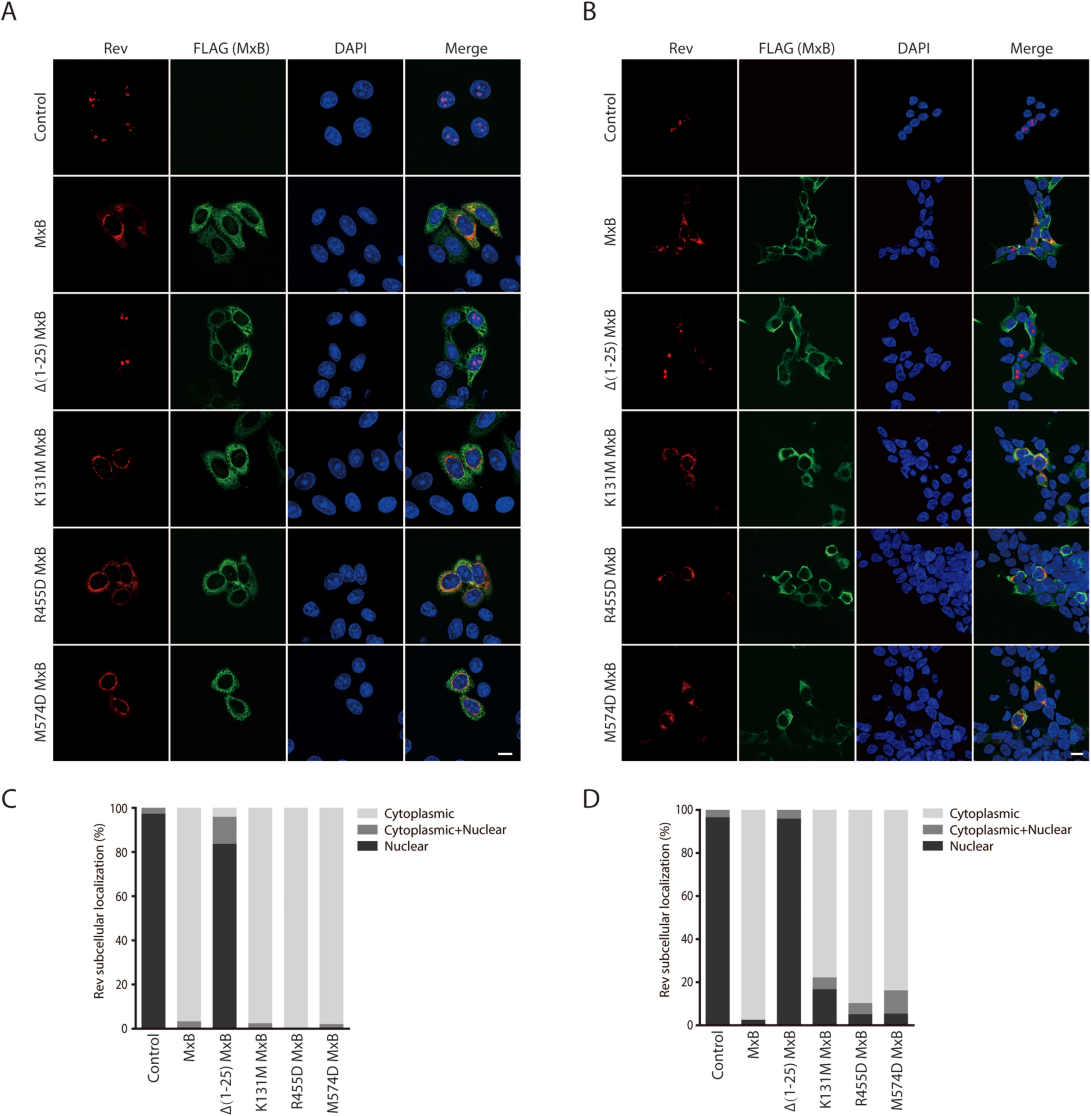
MxB inhibits Rev nuclear localization. Effect of MxB and its mutants Δ(1-25), K131M, R455D and M574D on the subcellular localization of Rev-RFP were determined in HeLa cells (A) and HEK293T cells (B). MxB-Flag and its mutants were detected with anti-Flag antibodies. The nuclei were stained with DAPI. Representative images are shown. Scale bar represents 10 µm. Cells with cytoplasmic, nuclear or both cytoplasmic and nuclear localizations of Rev were scored. For each transfection condition, 40–50 cells were examined. The percentage of cells with each phenotype was calculated, and the results are presented in (C) for HeLa cells, in (D) for HEK293T cells.

We asked whether, in addition to viral DNA, MxB also inhibits the nuclear import of viral proteins. Thus, we examined the effect of MxB on the subcellular localization of four HIV-1 proteins, Vpr (viral protein r), IN (integrase), Tat (transactivator of transcription) and Rev that are known to localize in the nucleus. The results of confocal microscopy showed that MxB changed the subcellular localization of Rev from the nucleus to the cytoplasm, while did not affect the nuclear localization of Vpr, IN and Tat proteins (Fig. S1). We further tested the effect of MxB and its mutants on Rev in HeLa and HEK293T cells, to ensure that the results are not cell line-dependent. The MxB mutants tested are defective in the key functional motifs and are impaired in HIV-1 inhibition to various degrees. The MxB mutant Δ1-25 lacks the first 25 amino acids and does not inhibit HIV-1 [11], K131M is defective in GTP binding yet capable of inhibiting HIV-1 infection [40], R455D and M574D are deficient in oligomerization and deficient in HIV-1 inhibition [14,15]. The results showed that the Δ1-25 MxB mutant, but not K131M, R455D, nor M574D, lost the ability of retaining Rev in the cytoplasm (Figure 1), suggesting the key role of the first 25 amino acids in MxB inhibition of Rev nuclear import.

### MxB inhibits Rev-dependent expression of HIV-1 Gag protein

**Figure 2.**
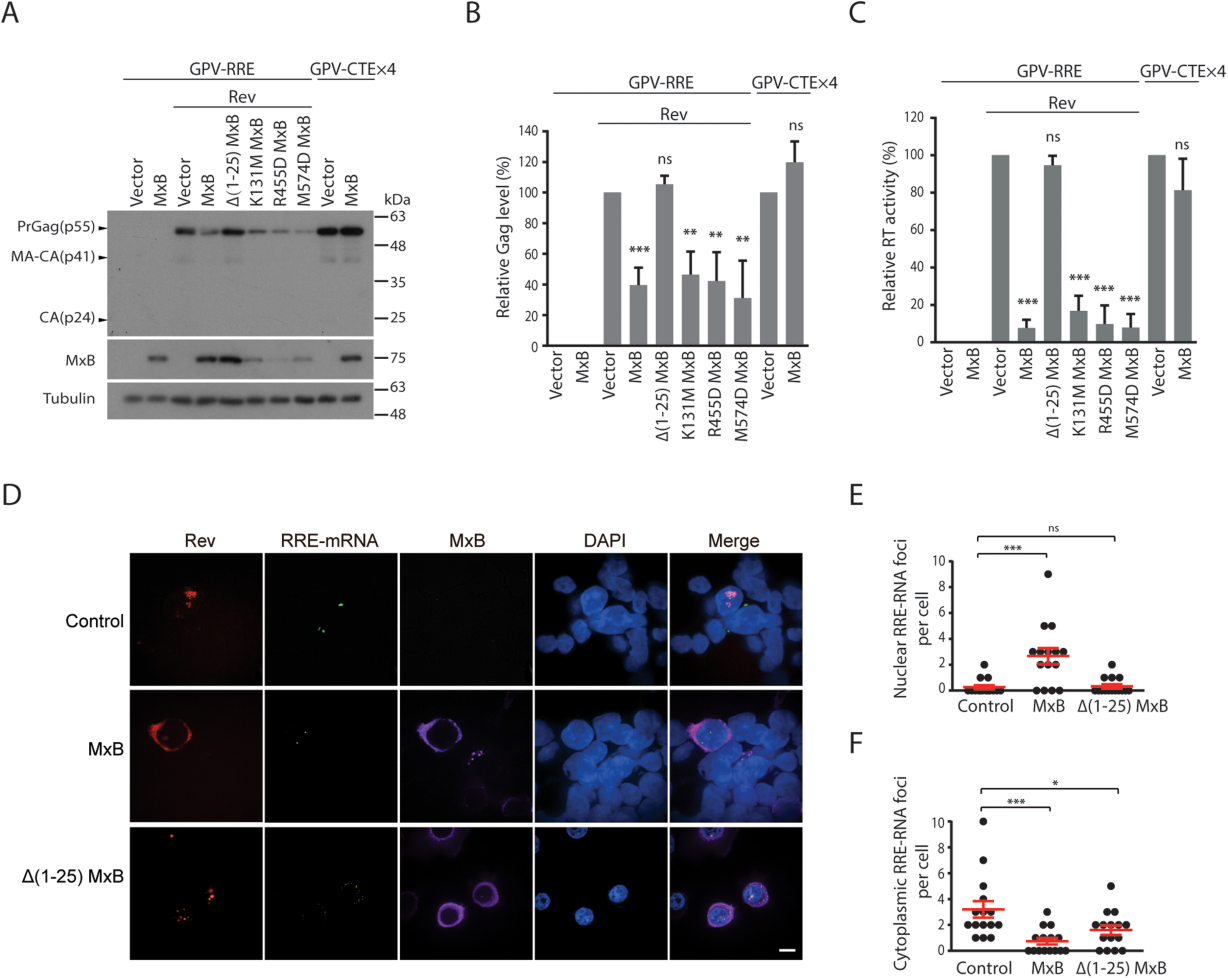
MxB inhibits Rev-dependent Gag expression. (A) MxB-Flag and its mutated DNA were transfected into HEK293T cells together with GPV-RRE and Rev. Expression of Gag, MxB and Tubulin in cell lysates were determined by Western blotting. Transfection experiment was also performed with GPV-CTE-x4 and MxB-Flag to examine the effect of MxB on Rev-independent Gag expression. Protein markers (kDa) are shown on the right side of the gels. (B) Levels of viral Gag proteins in the Western blots were quantified with Image J, the results from three independent experiments are presented in the bar graph. (C) Levels of viral RT activity in the culture supernatants were determined to measure the levels of VLPs. The values from the vector controls are arbitrarily set as 100. Results shown are the average of three independent transfection experiments. (D) Fluorescent in situ RNA hybridization to detect subcellular localization of the GPV-RRE RNA in the control and MxB-expressing cells. The cytoplasmic GPV-RRE RNA foci and nuclear GPV-RRE RNA foci per cell were counted, and the results are shown in (E) and (F). *P* values were calculated with reference to the vector control. Scale bar represents 10 µm. * indicates *p *<* *0.05. ** indicates *p *<* *0.01. *** indicates *p *<* *0.001. ns, not significant.

Next, we investigated whether, by sequestering Rev within the cytoplasm, MxB decreases Rev-mediated expression of HIV-1 Gag protein. Similar to viral Env protein, Gag is the main structural protein of HIV-1 particles, and its expression is dependent on Rev, as opposed to viral proteins such as Nef whose expression is Rev-independent. Two Gag-expression vectors were used in the study. GPV-RRE (GPV, Gag-Pol vector) carries the Rev response element (RRE), can only express Gag/Gag-Pol proteins in the presence of Rev, whereas GPV-CTEx4 bears four copies of the constitutive transport element (CTE) from Mason-Pfizer monkey virus, can express Gag/Gag-Pol independently of Rev [34]. MxB and its mutants were co-expressed with either of these two vectors, levels of Gag protein in the cell lysates were determined by Western blotting. The results showed marked decrease of Gag expression from GPV-RRE/Rev in cells expressing MxB but not its mutant Δ1-25 MxB (Figure 2(A,B)). This strong inhibition by MxB was further corroborated by the profound reduction of virus-like particles (VLPs, formed by Gag and Gag/Pol proteins) in the culture supernatants, as determined by the level of viral reverse transcriptase activity which is an integral part of viral Gag-Pol protein and associated with virus particles (Figure 2(C)). The observed inhibition is specific for Rev, since MxB did not affect Gag expression (Figure 2(A,B)) nor VLP production (Figure 2(C)) from the GPV-CTEx4 vector. We also transfected the GPV vectors and MxB DNA into Jurkat cells and observed that MxB decreased the Rev-mediated Gag expression from GPV-RRE by 2-fold, whereas observed no effect of MxB on Gag expression from GPV-CTEx4 (Fig. S2). In agreement with their abilities to retain Rev within the cytoplasm (Figure 1), MxB mutants K131M, R455D and M574D also strongly inhibited Rev-dependent Gag expression (Figure 2(A,B)) and VLP production (Figure 2(C)). The principal function of Rev is to export the RRE-containing HIV-1 RNA into the cytoplasm for translation [41]. We thus performed fluorescent in situ hybridization to detect the subcellular localization of GPV-RRE RNA, and observed marked nuclear sequestration of GPV-RRE RNA in MxB-expressing cells as opposed to the cytoplasmic localization of the majority of GPV-RRE RNA in the control cells and the Δ(1-25)MxB-expressing cells (Figure 2(D,E,F)), suggesting that MxB diminishes Rev-dependent RNA export. Together, we conclude that MxB impedes nuclear import of HIV-1 Rev protein, and as a result, inhibits Rev-dependent Gag expression.

### MxB disrupts the interaction of Rev protein with TNPO1

**Figure 3.**
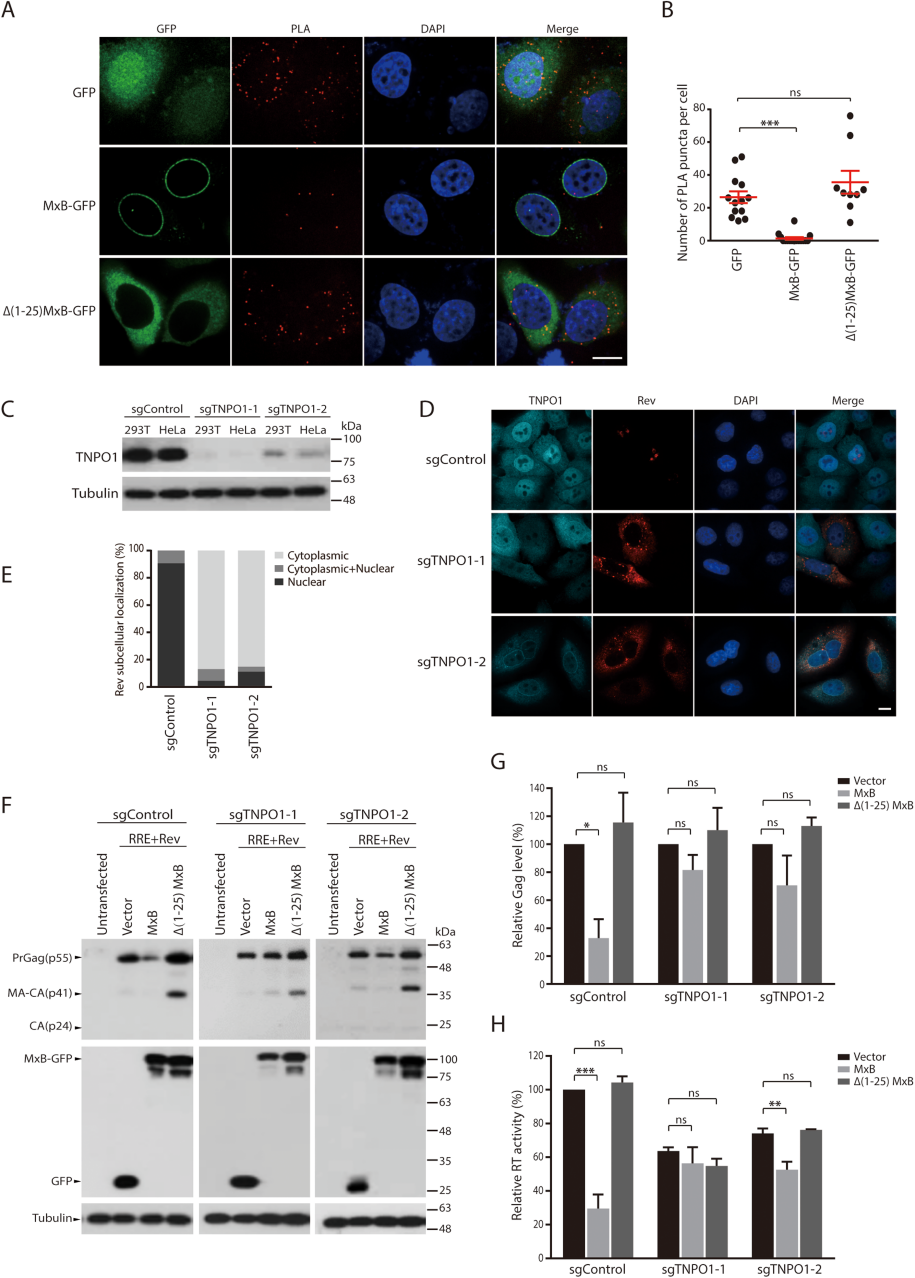
Effect of MxB on the association of Rev with TNPO1. (A, B) HeLa cells were transfected with Rev together with the GFP vector, MxB-GFP or Δ(1-25) MxB-GFP DNA. The association of Rev with TNPO1 was examined with PLA, and detected as red puncta in cells. Images of one representative experiment are shown in (A). The red puncta per cell from multiple cells of each transfection condition were scored, the results are presented in (B). (C) Western blots of TNPO1 expression in HEK293T or HeLa cells that were stably transduced with Cas9 and sgControl, sgTNPO1-1 or sgTNPO1-2. (D) Subcellular localization of Rev-RFP in HeLa cells with TNPO1 knockdown with Cas9/sgTNPO1. (E) Subcellular localization of Rev in 40 cells of either control or TNPO1 knockdown was determined, and the percentage results are presented in the bar graph. (F) Rev-dependent expression of Gag protein in HEK293T cells that were depleted of TNPO1 with Cas9/sgTNPO1. (G) Levels of Gag in the Western blots were determined with Image J, and the results of three independent experiments are shown in the bar graph. (H) Levels of viral RT activity in the supernatants of transfected HEK293T cells. The RT values from the Cas9/sgControl cells, which were transfected with GPV-RRE and Rev DNA, are arbitrarily set as 100. Results shown are the average of three independent transfection experiments. * indicates *p *<* *0.05. ** indicates *p *<* *0.01. *** indicates *p *<* *0.001. ns, not significant.

MxB has been shown to associate with TNPO1 [31], and TNPO1 can direct the nuclear import of Rev [42]. We thus speculated that MxB may impair the nuclear import of Rev through disrupting the interaction of TNPO1 and Rev. To test this, we performed the proximity ligation assay (PLA) to measure TNPO1 and Rev association, and observed the puncta signals in the cytoplasm and the nucleus (Figure 3(A,B)). Strong association of Rev with TNPO1 was observed, as measured by 26 puncta per cell on average, with majority of the signals within the cytoplasm (Figure 3(A,B)). However, expression of MxB, but not its Δ1-25 mutant that did not affect nuclear localization of Rev (Figure 1), nearly abrogated the association of Rev with TNPO1 (Figure 3(A,B)). To further determine the role of TNPO1 in MxB inhibition of HIV-1 Rev function, we used Cas9/sgRNA to deplete endogenous TNPO1 and examined the subcellular localization of Rev and Rev-dependent Gag expression in MxB-expressing cells. Two sgRNAs were tested, and both markedly diminished TNPO1 expression (Figure 3(C)). TNPO1 knockdown led to cytoplasmic retention of Rev (Figure 3(D,E)), diminished Rev-dependent expression of HIV-1 Gag protein (Figure 3(F,G)) and the production of VLPs (Figure 3(H)). Importantly, depletion of TNPO1 eliminated the inhibitory effect of MxB on Rev-dependent expression of Gag protein (Figure 3(F,G)) and VLP production (Figure 3(H)). HIV-1 Rev shuttles between the cytoplasm and the nucleus, and its nuclear export requires CRM1 (chromosomal maintenance 1, also known as exportin 1) [41]. We therefore used the CRM1 inhibitor leptomycin B (LMB) to block CRM1-dependent export of Rev and asked whether MxB can still retain Rev within the cytoplasm. The results of Figure S3 showed that LMB treatment led to nuclear localization of Rev in MxB-expressing cells, suggesting that MxB may not completely block the nuclear import of Rev, thus when CRM1-dependent nuclear export is occluded by LMB, Rev accumulates within the nucleus. Together, these data demonstrate that MxB inhibits the nuclear import of Rev by disrupting the association of Rev with TNPO1.

### TNPO1-independent Rev variants resist MxB inhibition

**Figure 4.**
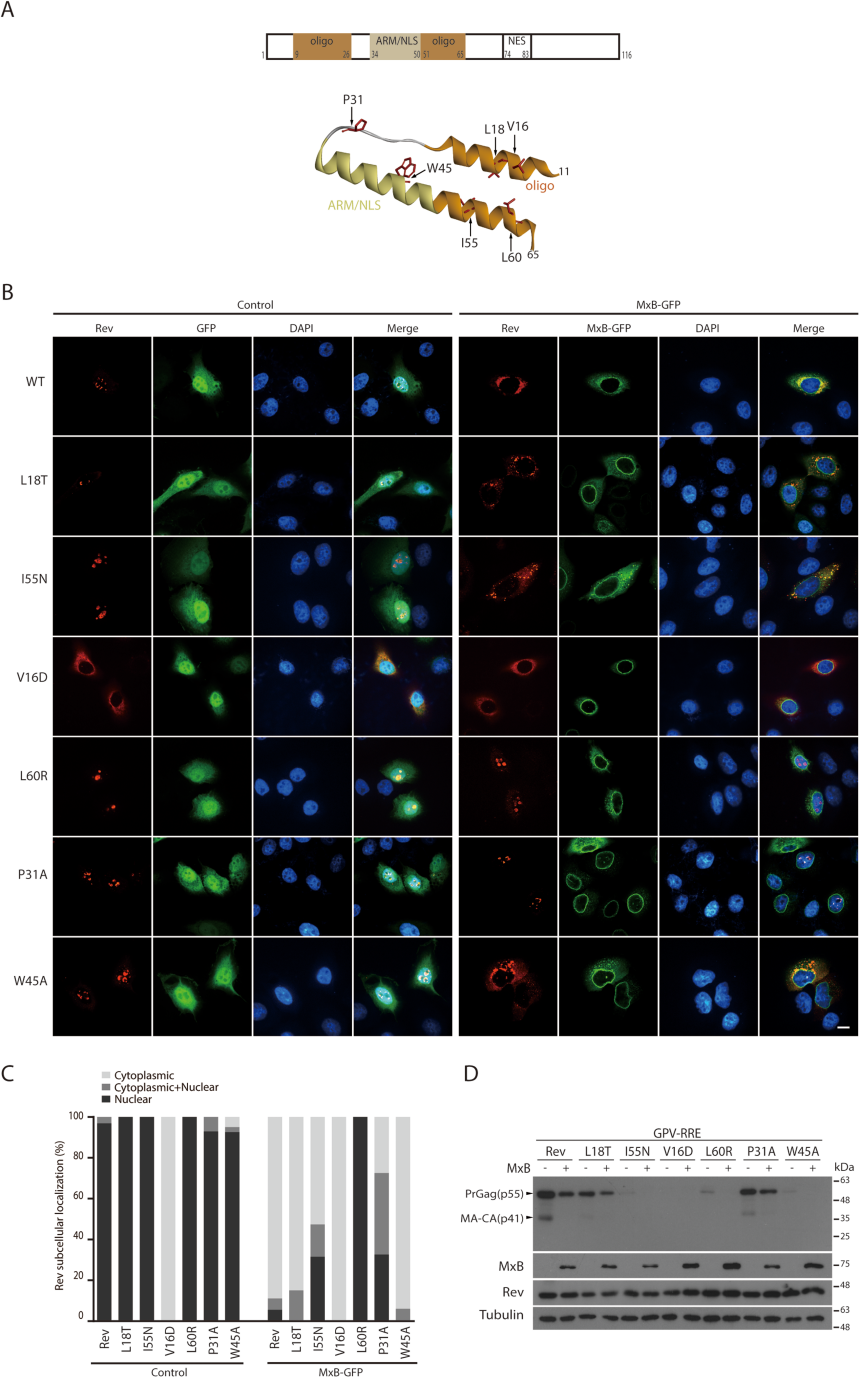
Identification of MxB-resistant Rev mutants. (A) Illustration of Rev mutations in the context of Rev structure. Shown at the top are Rev domains, oligomerization domain, ARM (arginine rich motif)/NLS (nuclear localization signal), and NES (nuclear export signal). Amino acid positions of each domain are also denoted. (B) Subcellular localization of Rev-RFP and its mutants in HeLa cells expressing GFP or MxB-GFP. Representative images are shown. Scale bar represents 10 µm. (C) Subcellular localization of Rev mutants was determined in 40 cells under each condition, and the results are presented in the bar graph. (D) Western blots of Gag expression mediated by Rev or its mutants from the GPV-RRE vector. Results shown represent three independent transfection experiments.

We next asked whether there exist Rev mutations that resist MxB inhibition. We started by testing a group of Rev mutations that have been previously characterized for their ability of affecting Rev multimerization and Rev’s function in HIV-1 gene expression [43–45]. Locations of these mutations in the domain structure of Rev are shown in Figure 4(A). Mutations V16D, L18T, W45A, I55N, and L60R are located in the α-helices, P31A in the linker region. We postulated that some of these Rev mutants may respond differently to MxB compared to the wild type Rev, thus providing an opportunity to further investigate the mechanism underpinning MxB sensitivity. Results of confocal microscopy showed that similar to the wild type Rev protein, Rev mutants L18T, P31A, W45A, I55N and L60R were located within the nucleus, whereas the V16D mutant was predominantly cytoplasmic (Figure 4(B,C)). In MxB-expressing cells, localization of the L18T and I55N mutants changed from the nucleus to the cytoplasm. In contrast, the P31A and L60R mutants were still located in the nucleus in the presence of MxB (Figure 4(B,C)), suggesting their resistance to MxB inhibition. Among these six Rev mutants, V16D, W45A, I55N, and L60R were severely impaired in stimulating Gag expression, L18T was moderately impaired and P31A performed as the wild type Rev (Figure 4(D)). Since the P31A Rev remains nuclear in MxB-expressing cells, we further investigated the activity of P31 in resisting MxB.

**Figure 5.**
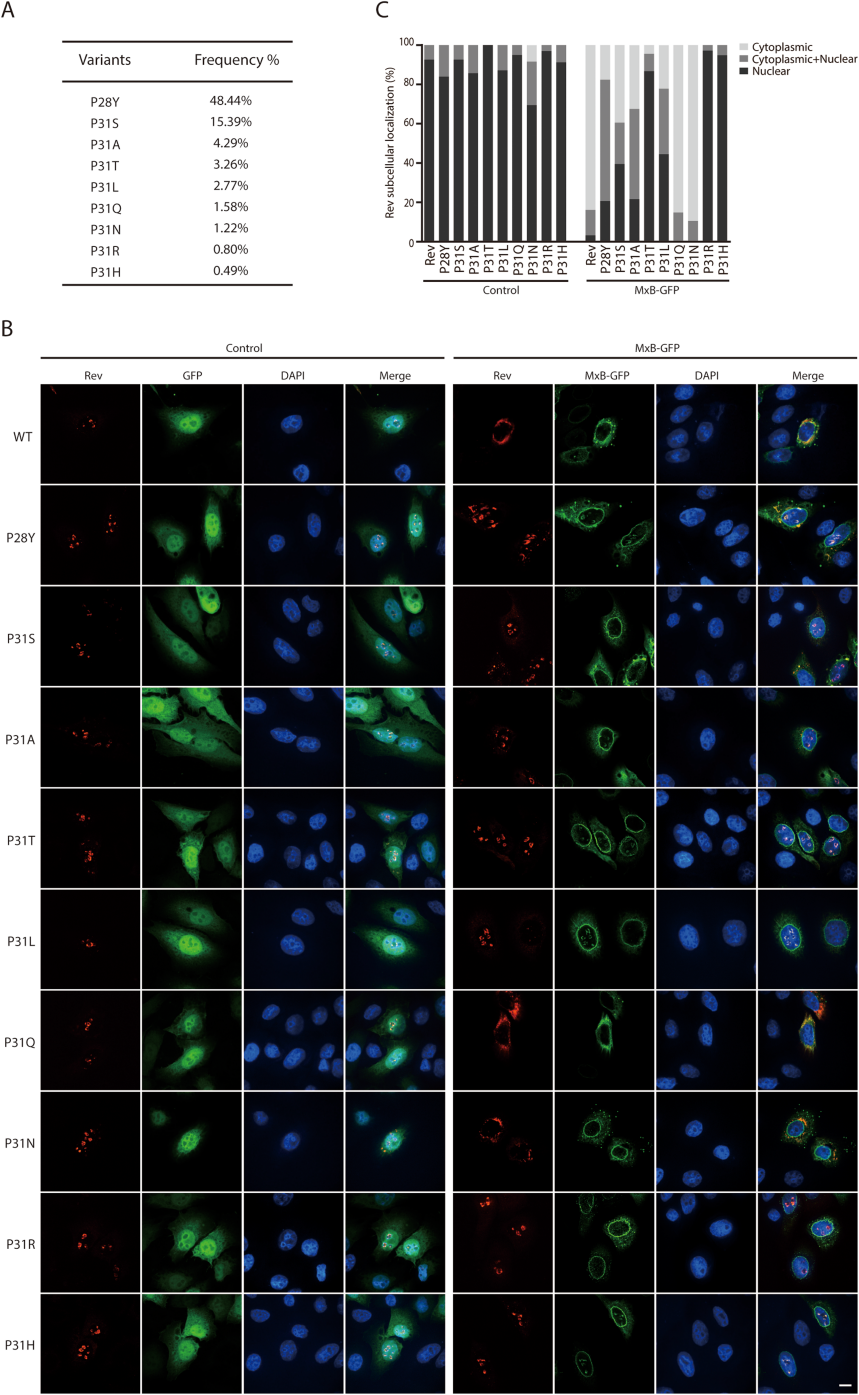
Nuclear localization of Rev variants in MxB-expressing cells. (A) Frequencies of Rev variants at amino acid position P31 in the circulating HIV-1 strains (https://www.hiv.lanl.gov/content/index). (B) Subcellular localization of Rev variants in HeLa cells expressing GFP or MxB-GFP. Representative images are shown. Scale bar represents 10 µm. (C) Cells were scored for having only nuclear Rev, only cytoplasmic Rev, with both nuclear and cytoplasmic Rev. 40 cells were examined for each transfection condition. The results are shown in the bar graph.

When we inspected Rev sequences in the HIV-1 strains that were isolated from patients (https://www.hiv.lanl.gov/content/index), we found a high degree of polymorphism at position P31 (Figure 5(A)). We thus further tested Rev variants of P31 for their resistance to MxB inhibition, these include P31A, P31S, P31T, P31L, P31Q, P31N, P31R and P31H (Figure 5(A)). We also included in the study the P28Y variant which has a 48.44% frequency and is located close to P31. All these Rev variants except P31N were seen exclusively within the nucleus (Figure 5(B)). When MxB was expressed, the P31Q and P31N mutants were sequestered within the cytoplasm in a fashion similar to that of wild type Rev, while the other Rev variants showed different degrees of nuclear localization, with P31R and P31H showing complete resistance to MxB inhibition of nuclear import, followed by P31T, P31L, P31S, P31A and P28Y, ranked by their decreased nuclear localization in MxB-expressing cells (Figure 5(B,C)). Therefore, in HIV-1 strains from patients, there exist Rev variants that resist MxB inhibition of nuclear localization.

### MxB-resistant Rev variants are independent of TNPO1 in nuclear localization

**Figure 6.**
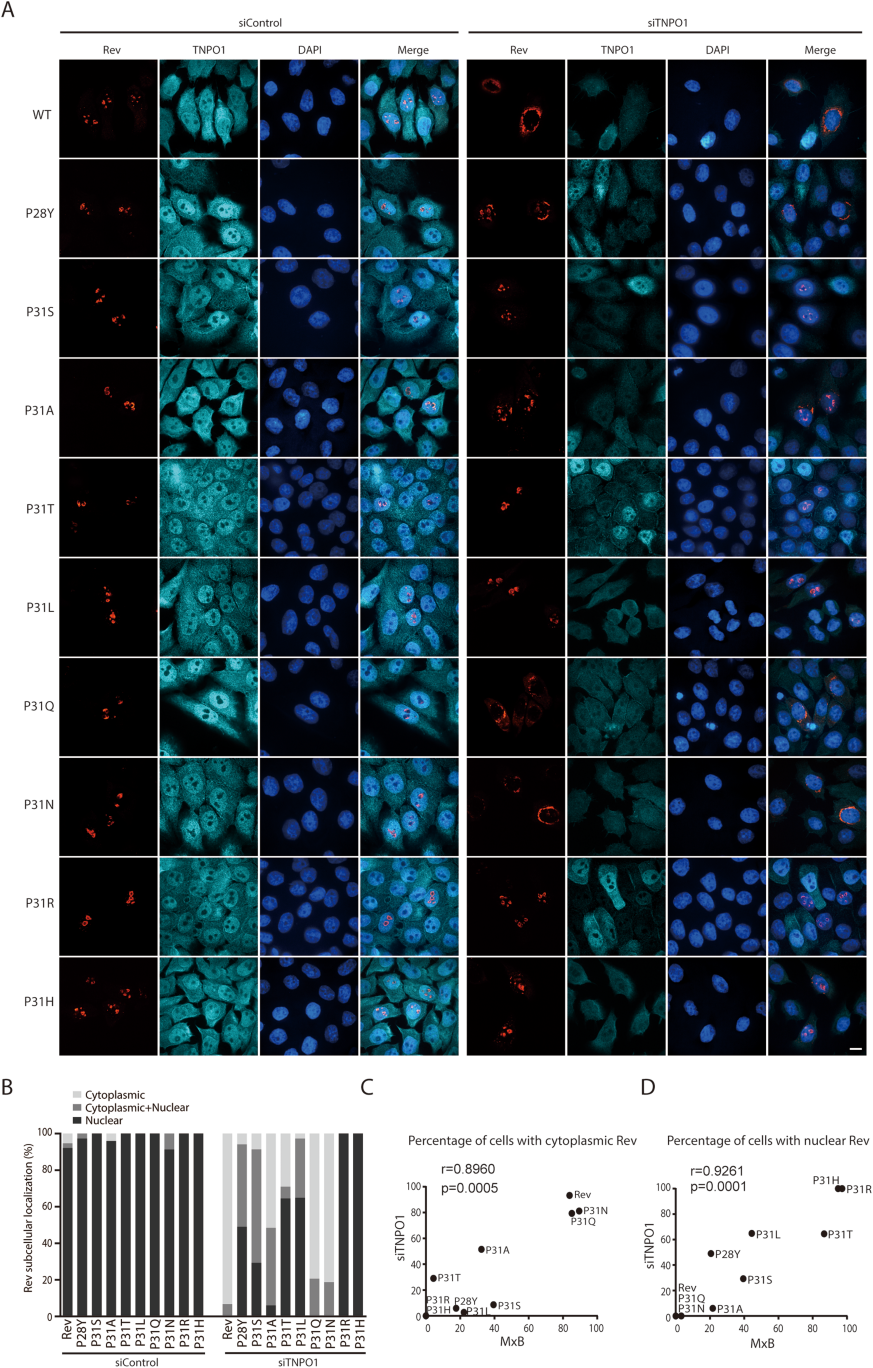
Subcellular localization of Rev and its variants in TNPO1 knockdown cells. (A) Rev-RFP or Rev variants were expressed in HeLa cells that were transfected with control siRNA or TNPO1-targeting siRNA. TNPO1 was detected with anti-TNPO1 antibodies. Representative images are shown. (B) Cells were scored for having only nuclear Rev, only cytoplasmic Rev, or both cytoplasmic and nuclear Rev. 50 cells were examined for each transfection condition. The results are presented in the bar graph. (C, D) Sensitivity of subcellular localization of Rev variants to TNPO1 knockdown correlates with their sensitivity to MxB inhibition. This correlation was calculated either using the percentages of TNPO1 knockdown cells with cytoplasmic Rev (TNPO1-dependent) and the percentages of MxB-expressing cells with cytoplasmic Rev (MxB-sensitive) (shown in (C)), or using the percentages of TNPO1-knockdown cells with nuclear Rev (TNPO1-independent) and the percentages of MxB-expressing cells with nuclear Rev (MxB-resistant) (shown in (D)). The Pearson correlation coefficient (Pearson’s *r*) was calculated with GraphPad Prism.

Since MxB disrupts the association of Rev with TNPO1 (Figure 3(A,B)), we suspected that the MxB-resistant Rev variants may enter the nucleus independently of TNPO1. To test this, we knocked down TNPO1 and examined the subcellular localization of Rev and its variants. Not surprisingly, loss of TNPO1 led to cytoplasmic retention of wild type Rev (Figure 6(A,B)). The Rev variants showed a spectrum of responses to TNPO1 depletion. While P31Q and P31N behaved like the wild type Rev, and were blocked within the cytoplasm as a result of TNPO1 knockdown, the P31R and P31H mutants remained completely nuclear, with P31L, P31T, P28Y, P31S and P31A exhibiting gradually decreased nuclear localization with TNPO1 knockdown (Figure 6(A,B)). Therefore, P31R and P31H are independent of TNPO1 in nuclear import, and coincidently, also resist MxB inhibition. We took advantage of the different degrees of sensitivities of Rev variants to TNPO1 knockdown and to MxB inhibition, and calculated how strong the correlation is between the resistance to MxB inhibition and the independence of TNPO1 among these Rev variants. To calculate the Pearson correlation coefficient values, we used the percentages of MxB-expressing cells that had nuclear Rev variants to represent the degrees of resistance to MxB, and the percentages of TNPO1-depleted cells having nuclear Rev variants to represent the degrees of TNPO1 independence. The results showed a very strong correlation between these two functions with the Pearson’s r values of 0.8960 (*p* = 0.0005) and 0.9261 (*p* = 0.0001) (Figure 6(C,D)), further demonstrating that Rev is able to maintain nuclear localization in MxB-expressing cells through employing a TNPO1-independent nuclear import pathway. In addition to TNPO1, importin-β (IMP-β, also called karyopherin subunit beta 1, KPNB1) is another major nuclear import factor. We thus knocked down IMP-β with siRNA oligos to investigate whether the TNPO1-independent Rev variants use IMP-β for nuclear import. No effect on the nuclear localization of wild type Rev and its variants was observed (Fig. S4), indicating that the TNPO1-independent Rev variants enter the nucleus using importins other than IMP-β.

**Figure 7.**
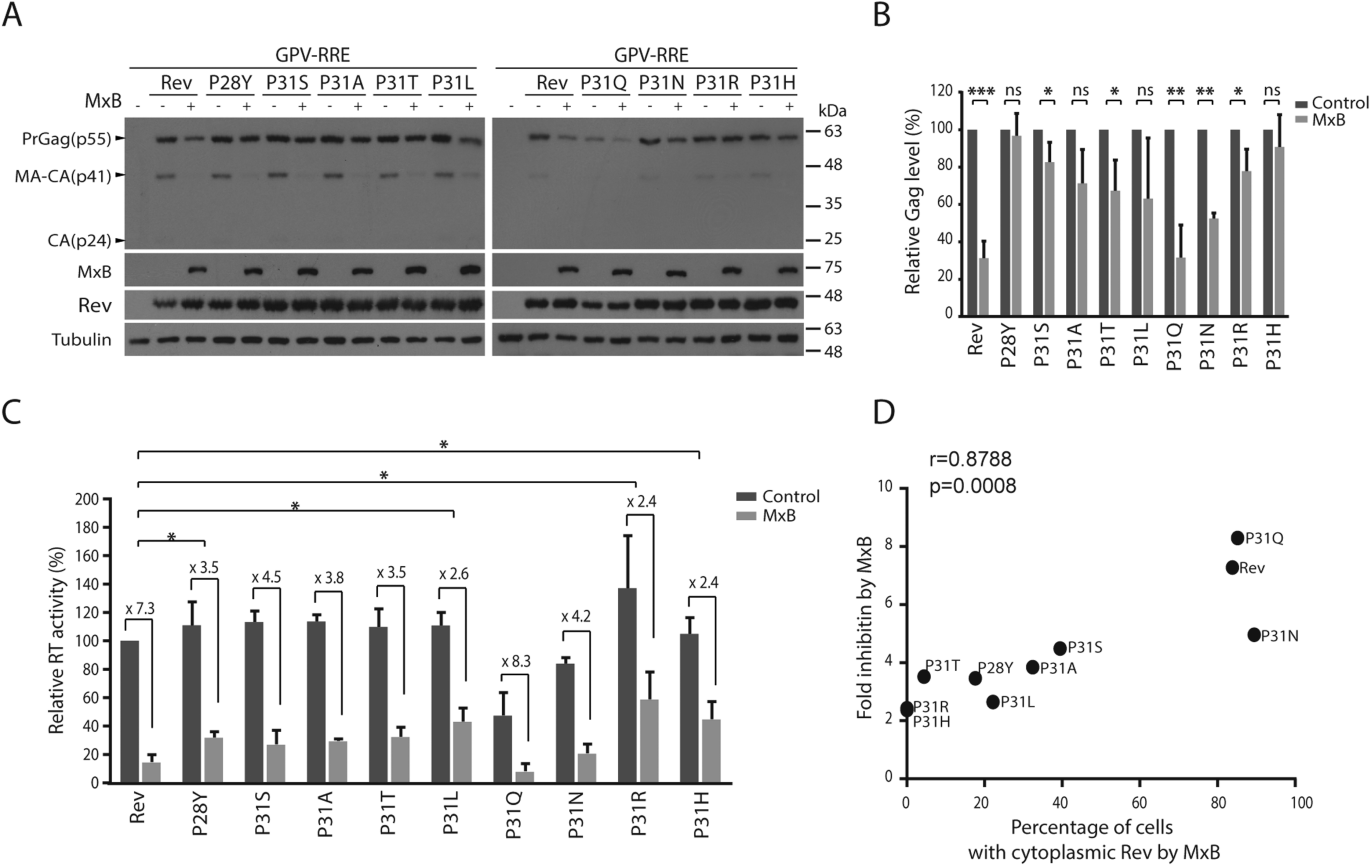
Some Rev variants are less restricted by MxB in mediating Gag expression. (A) Rev variants were co-transfected into HEK293T cells together with GPV-RRE and MxB DNA. Levels of Gag in the cell lysates were determined by Western blotting. (B) Levels of Gag in the Western blots were measured with Image J. The results of three independent experiments are shown in the bar graph. * indicates *p *<* *0.05. ** indicates *p *<* *0.01. *** indicates *p *<* *0.001. ns, not significant. (C) Viral RT activities in the supernatants of the transfected HEK293 T cells. RT values of the transfection with GPV-RRE, wild type Rev and vector DNA are arbitrarily set as 100. The averages of three independent experiments are presented. Fold of inhibition by MxB is shown for each Rev variant. The *p* values were calculated with reference to the fold of inhibition of wild type Rev by MxB. Statistically significant *p* values are denoted, with * indicating *p *<* *0.05. (D) Fold inhibition of RT levels by MxB (shown in (C)) correlates with percentages of MxB-expressing cells with cytoplasmic Rev (inhibition of Rev nuclear import by MxB, shown in Figure 6C). The Pearson correlation coefficient (Pearson’s *r*) was calculated with GraphPad Prism.

We posited that these TNPO1-independent Rev variants may efficiently mediate Gag expression in the presence of MxB. Indeed, in contrast to the strong inhibition of wild type Rev-mediated Gag expression and VLP production by MxB, much less inhibition was observed for the P31R and P31H Rev variants (Figure 7(A,B,C)). We further calculated the correlation between the folds of inhibition of VLP production by MxB (Figure 7(C)) and the percentages of MxB-expressing cells with cytoplasmic localization of the Rev variants (Figure 5(C)), and observed a significantly strong correlation, with a Pearson’s *r*-value of 0.8788 (*p* = 0.0008) (Figure 7(D)). Taken together, our data demonstrate that HIV-1 Rev protein is able to resist MxB inhibition through adopting TNPO1-independent nuclear import pathway.

### HIV-1 expresses higher levels of multiply spliced viral RNA and Rev protein to resist MxB inhibition

**Figure 8.**
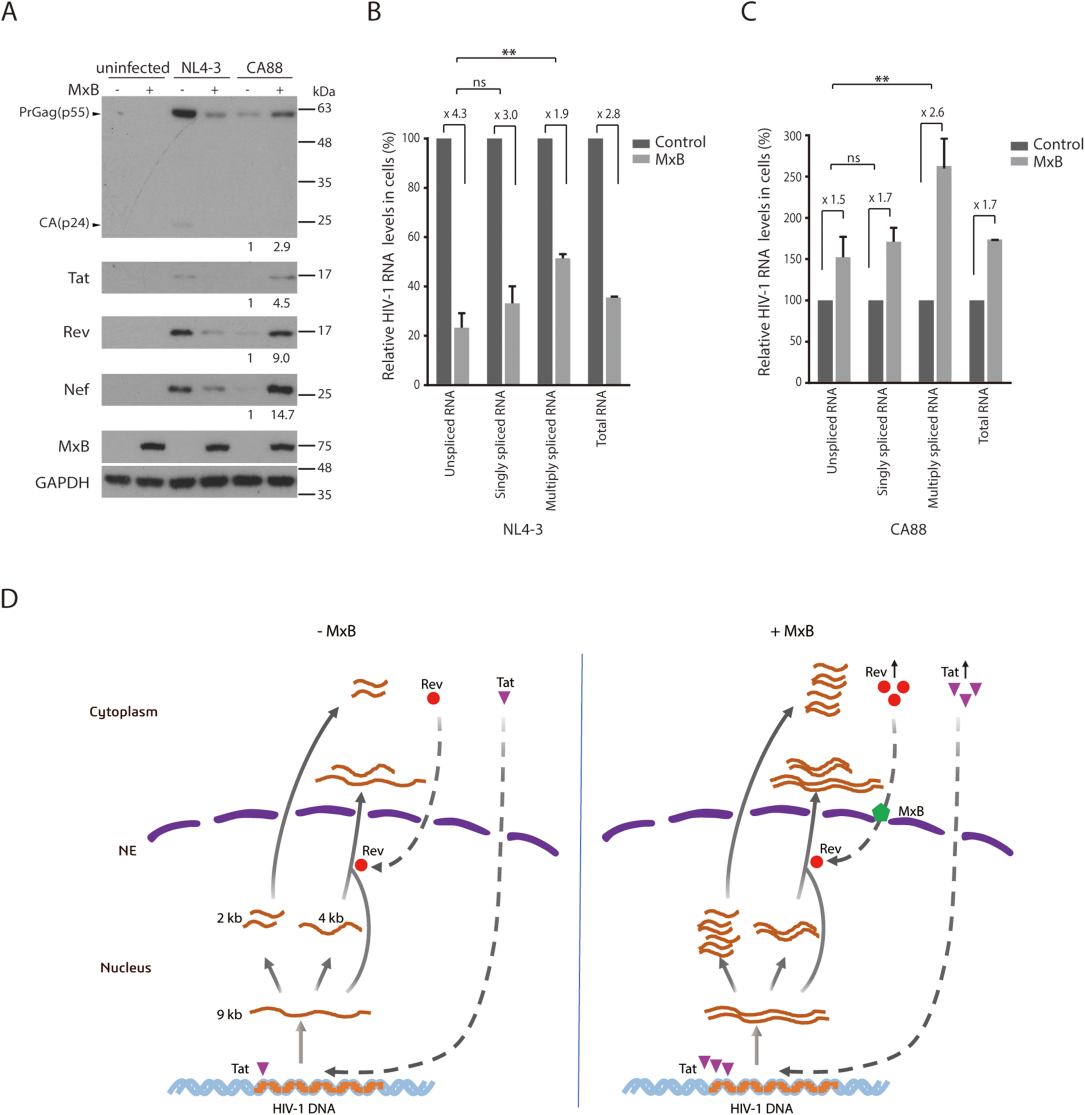
HIV-1 expresses more Rev protein in response to MxB inhibition. (A) Western blots of control or MxB-expressing SupT1 cells infected with HIV-1 NL4-3 or HIV-1 CA88. Levels of Gag/p24, Tat, Rev and Nef proteins were examined by Western blotting. GAPDH protein serves as the internal control. The signal intensities of protein bands were determined by Image J, and relative levels are presented. Results shown represent three independent infection experiments. (B, C) Relative levels of HIV-1 RNA in the infected SupT1 cells were determined by RT-qPCR. Results of HIV-1 NL4-3 and CA88 are shown in (B) and (C), respectively. Levels of unspliced RNA, singly spliced RNA, multiply spliced RNA, and total HIV-1 TAR were measured. The values of RT-qPCR from the infected control SupT1 cells are arbitrarily set as 100 for each viral RNA species. Results report the change of viral RNA in the infected MxB-expressing SupT1 cells relative to that in the infected control SupT1 cells. ** indicates *p *<* *0.01. ns, not significant. (D) A model to illustrate the potential effect of MxB on HIV-1 RNA expression. HIV-1 RNA expression is regulated by viral Tat and Rev proteins. Three species of HIV-1 RNA are produced, the unspliced full-length viral RNA (9 kb), singly spliced viral RNA (4 kb), and multiply spliced RNA (2 kb). Tat stimulates viral RNA transcription, Rev directs the export of 9 and 4 kb viral RNA into the cytoplasm. In the presence of MxB, nuclear import of Rev is inhibited. As a result, the 9 kb RNA cannot be exported into the cytoplasm, thus is spliced to form the 2 kb RNA which is exported into the cytoplasm independently of Rev and produces more Tat and Rev proteins. Tat continues to increase HIV-1 RNA synthesis, until Rev protein accumulates to a level high enough to overcome MxB inhibition. NE, nuclear envelope.

While some Rev variants are able to enter the nucleus in the presence of MxB, their function of stimulating Gag expression is still diminished by MxB to some degrees (Figure 7). We thus asked whether HIV-1 has other means to overcome MxB-mediated inhibition of Rev function. We infected the control and MxB-expressing SupT1 cells with HIV-1 NL4-3, and observed a sharp decrease of viral Gag expression in the MxB-expressing cells (Figure 8(A)), consistent with what we previously reported [6]. When the levels of viral RNA were measured by reverse transcription (RT) quantitative PCR (qPCR) as described in [39], the results showed that the level of unspliced and singly spliced HIV-1 RNA in the MxB-expressing SupT1 cells was 4.3- and 3.0-fold lower than that in the control SupT1 cells, respectively. However, the level of multiply spliced viral RNA decreased by only 1.9-fold (Figure 8(B)). It was difficult to determine whether the levels of Tat, Rev and Nef proteins, which are expressed from the multiply spliced viral RNA, were less affected by MxB compared with that of Gag which is expressed from unspliced viral RNA, because of the substantial reduction of these viral proteins by MxB, as shown in the Western blots (Figure 8(A)). We thus examined the infection of the HIV-1 CA88 virus which carries the A88T mutation in viral CA protein and resists MxB inhibition of viral DNA entry into the nucleus [6]. The A88T mutation diminished viral infection in the control SupT1 cells compared to the wild type HIV-1, and moderately increased viral infection in the MxB-expressing SupT1 cells, as shown by the results of Western blots (Figure 8(A)), which is in agreement with our previous report [6]. This increase in CA88 Gag expression in MxB-expression cells is not a result of an elevation in viral DNA integration [6], but rather a moderately higher level of total viral RNA expression (Figure 8(C)). When we quantified the levels of various CA88 viral RNA species by RT-qPCR, we observed a moderate 1.5- and 1.7-fold increase of unspliced and singly spliced CA88 viral RNA, and a marked 2.6-fold increase of multiply spliced viral RNA in MxB-expressing SupT1 cells, compared to those in the control cells (Figure 8(C)). When we measured the levels of CA88 viral proteins in the infected cells by Western blotting, in contrast to a moderate increase in viral Gag expression (2.9-fold), which corroborates the 1.5-fold increase in the unspliced viral RNA, a much greater increase of Tat (4.5-fold), Rev (9-fold) and Nef (14.7-fold) proteins in the MxB-expressing SupT1 cells was observed (Figure 8(A)). These data suggest that in response to the inhibition of Rev by MxB, HIV-1 increases the production of Rev protein to overcome MxB inhibition.

## Discussion

In this study, we report that MxB blocks the nuclear import of HIV-1 Rev protein by impairing the interaction of Rev with TNPO1. MxB has been shown to inhibit the infection of HIV-1 and herpesviruses by preventing the nuclear import of viral DNA [4–6,8–10]. Several mechanisms have been put forward to illuminate this function of MxB. One is the association of MxB with viral capsid [14,46]. HIV-1 capsid is involved in the docking of viral DNA onto the nuclear pore complex (NPC) and the entry of viral DNA into the nucleus [47]. And these functions of capsid can be affected by the associated MxB protein. A second mechanism is related to the localization of MxB to the nuclear pore complex [48]. With this, MxB may modulate the function of certain nucleoporins that are key to the nuclear import of HIV-1 DNA [30,31]. Also, MxB may affect the activity of certain nuclear transport receptors and thus modulate the nuclear import of the related cargos. This last mechanism was supported by the interaction of MxB with TNPO1, reported by Dicks et al [31], and is further supported by our data showing that MxB disrupts the interaction of TNPO1 with Rev, thus causing retention of Rev within the cytoplasm.

Results of our mutagenesis experiments showed that the N-terminal sequence of MxB is essential to the inhibition of Rev nuclear import, but the active GTPase domain as well as protein oligomerization appear to be dispensable for this inhibition. This essential role of the N-terminal sequence has also been reported in MxB inhibition of the nuclear import of HIV-1 and herpesvirus DNA [4,8,9,11]. In addition, protein oligomerization is also required for MxB to inhibit nuclear import of viral DNA [9,14,15,18,19], which may reflect the need of MxB to efficiently associate with the complex structure of viral capsid in order to exert its inhibitory effect [14,15,22,23]. The GTPase activity is needed for effectively inhibiting herpesvirus but not for inhibiting HIV-1 [4,5,8,9]. These findings suggest that MxB may deploy different combinations of its domains and functions to block the nuclear import of protein assemblies that have different levels of complexity and ride along different import pathways.

HIV-1 Rev protein bears an arginine-rich nuclear localization signal (NLS) which has been shown to bind to IMP-α or directly to IMP-β, and directs the nuclear localization of Rev [41,49,50]. In addition, depleting TNPO1 has been shown to cause cytoplasmic retention of Rev [42], which was also observed in our study. However, a PY-NLS (PY, a proline-tyrosine motif), which is recognized by TNPO1, has not been identified in Rev. It is possible that TNPO1 binds to a non-canonical NLS in Rev and transports Rev into the nucleus. Nonetheless, we identified Rev variants that enter the nucleus independently of TNPO1. And the nuclear localization of these Rev variants is not affected by MxB. This result further supports the mechanism that MxB impairs Rev nuclear import by targeting and disrupting Rev utilization of TNPO1. Since some of these Rev variants exist in the circulating HIV-1 strains, it is possible that HIV-1 Rev acquires and preserves these mutations to resist MxB inhibition.

We currently do not know whether MxB also affects the nuclear import of other TNPO1 cargos. Using the light inducible nuclear export live imaging system [36], we tracked the nuclear import of the mCherry reporter protein bearing either the c-Myc NLS or the PY-NLS M9 in control and MxB-expressing cells. We did not observe any notable effect from MxB on the nuclear import kinetics of these reporter proteins (Fig. S5). It appears that rather than exerting a general impact on protein entry into the nucleus, MxB may affect the nuclear import of a selective group of proteins. In support of this possibility, Kane et al reported the inhibitory effect of MxB on the nuclear localization of reporter proteins bearing NLS motifs from c-Myc, DDX21 (DEAD-box helicase 21), hnRNP K (heterogeneous nuclear ribonucleoprotein K), and MxB itself [30]. More MxB-targeted viral and cellular proteins need to be identified before we can discern the pattern and illuminate the underlying mechanisms. Since some of the TNPO1 cargos, such as hnRNP A1 [51,52], have been reported to modulate HIV-1 gene expression, in addition to impairing the function of viral Rev protein, MxB may inhibit the expression of HIV-1 RNA and proteins also by deterring the nuclear import of such TNPO1 cargo proteins.

Virus evolution experiments in cultured cells have selected MxB-resistant HIV-1 that carries mutations in viral CA protein [6,28], no mutations in Rev protein were reported in these studies. Then how has HIV-1 managed to overcome the inhibition of Rev by MxB in these studies? One mechanism is that HIV-1 can express more Rev protein, as shown by our results (Figure 8). This mechanism is a direct result of the feedback regulation in HIV-1 gene expression [53], as illustrated in Figure 8(D). At the very beginning of HIV-1 RNA expression, the newly synthesized full-length viral RNA quickly undergoes splicing to become completely spliced RNA species which are exported into the cytoplasm to produce viral Tat, Rev and Nef proteins. Tat and Rev proteins then enter the nucleus. Tat strongly stimulates HIV-1 RNA transcription, Rev directs the nuclear export of the full-length and the singly spliced viral RNA that bear the RRE motif. An equilibrium will be reached between the rate of viral transcription (controlled by the level of Tat protein), the rate of nuclear export of full-length and singly spliced viral RNA (controlled by the level of Rev protein), and the rate of viral RNA splicing (controlled by cellular spliceosome and accessary proteins). When Rev function is inhibited by MxB, the newly synthesized full-length HIV-1 RNA cannot be exported from the nucleus to the cytoplasm, thus is completely spliced into the 2-kb RNA molecules to express more Tat, Rev and Nef. More Tat then further enhances viral RNA synthesis, until the level of Rev is high enough to overcome the inhibition by MxB and begins to export unspliced and singly spliced viral RNA into the cytoplasm.

In conclusion, our study demonstrates that MxB inhibits nuclear import of HIV-1 Rev protein, thus extending the antiviral mechanisms of MxB from targeting the nuclear import of viral DNA to also targeting the nuclear import of essential viral proteins. Furthermore, the inhibitory action of MxB may be attributed to its interference of the engagement of TNPO1 by viral or cellular proteins for nuclear import. Importantly, HIV-1 resists the inhibition of Rev by MxB through evolving TNPO1-independent Rev protein and increasing Rev expression.

## Supplementary Material

Fig_S5.tif

Fig_S4.tif

Fig_S3.tif

Fig_S2.tif

Fig_S1.tif
