# Supplementary figures and images for: HIV-1 resists MxB inhibition of viral Rev protein
Source: Emerg Microbes Infect. 2020 Sep 20;9(1):2030–45. doi: 10.1080/22221751.2020.1818633 (PMC7534208; doi:10.1080/22221751.2020.1818633)

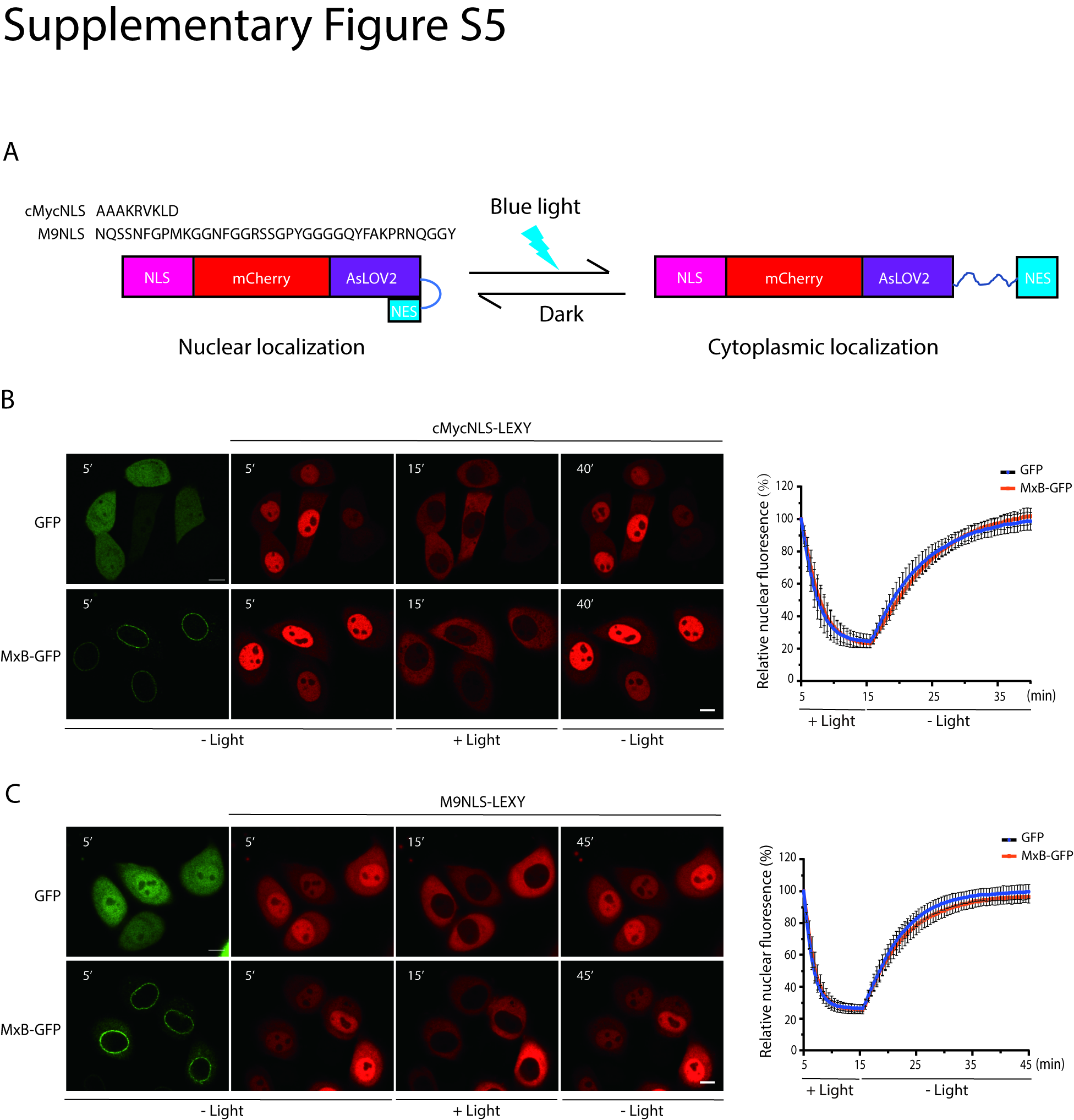

Supplement: Fig_S5.tif [file TEMI_A_1818633_SM7209.tif]

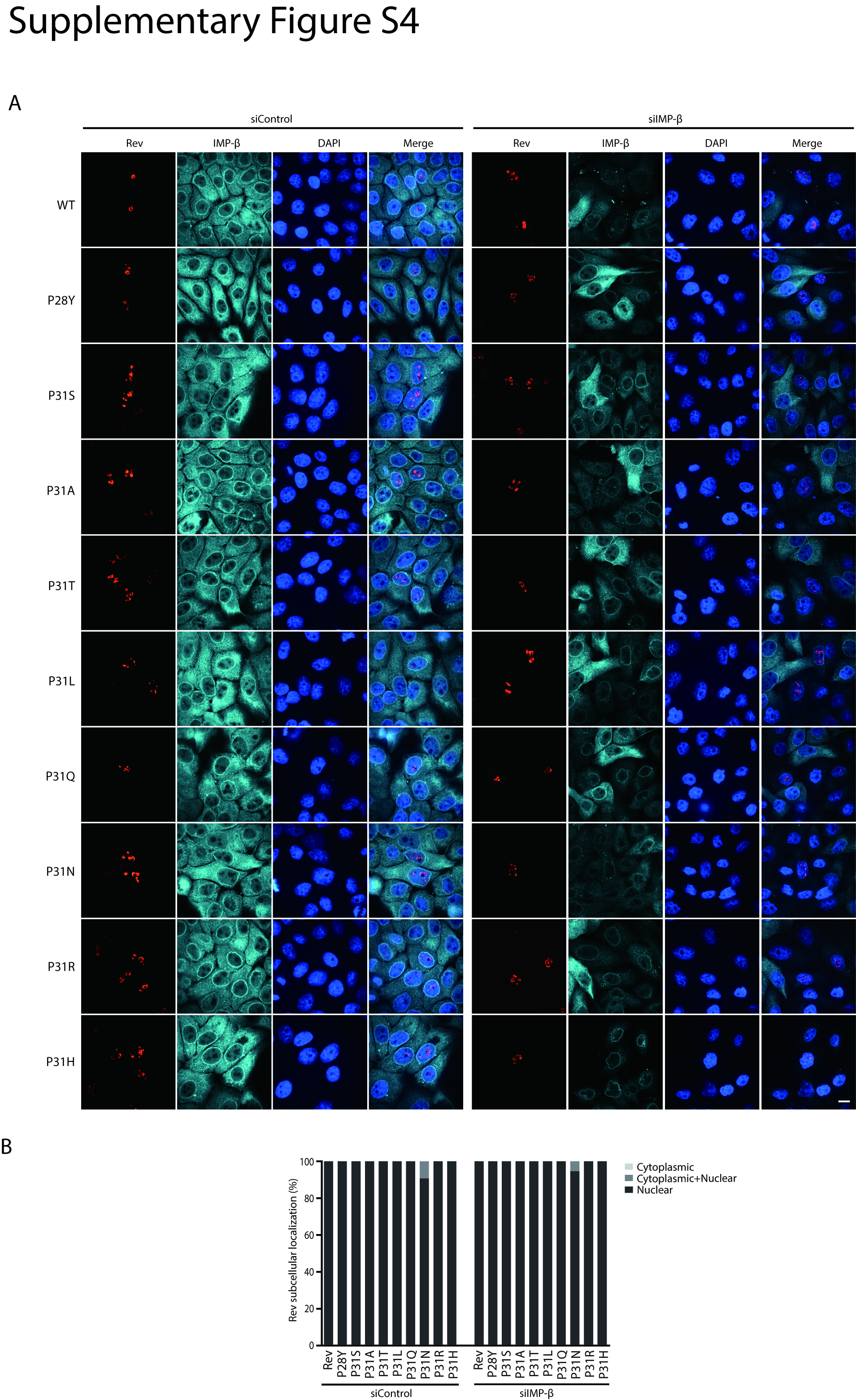

Supplement: Fig_S4.tif [file TEMI_A_1818633_SM7208.tif]

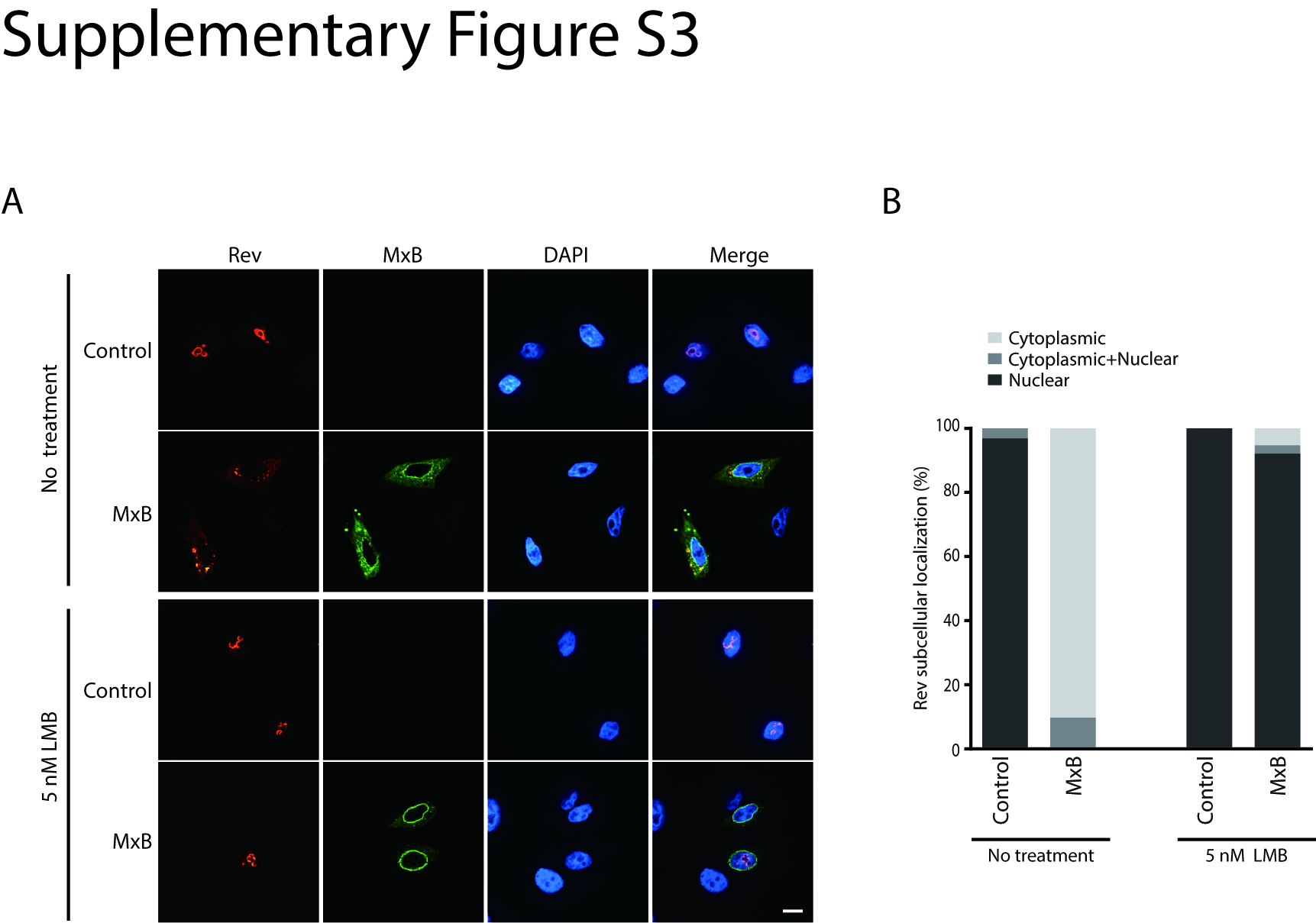

Supplement: Fig_S3.tif [file TEMI_A_1818633_SM7207.tif]

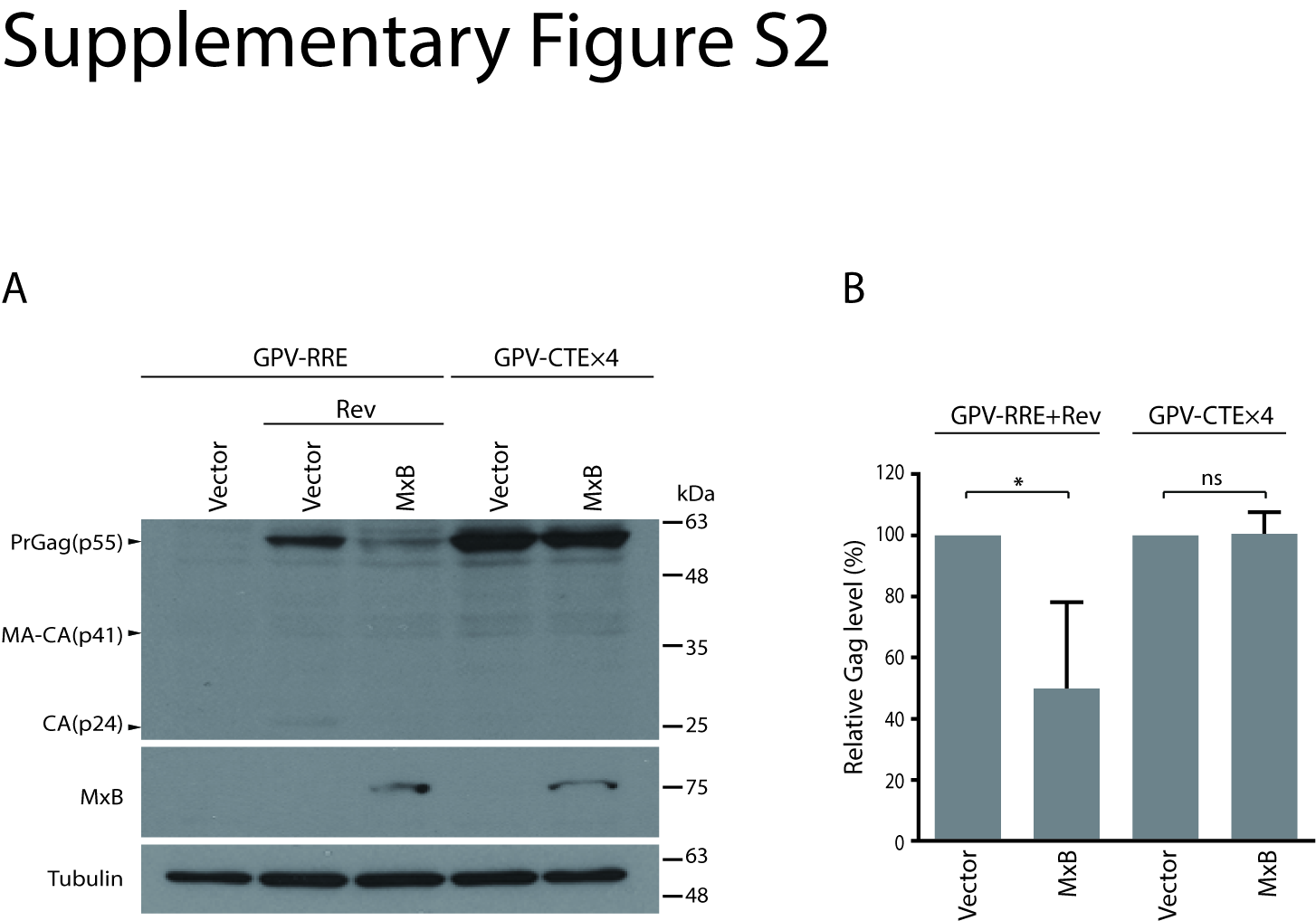

Supplement: Fig_S2.tif [file TEMI_A_1818633_SM7206.tif]

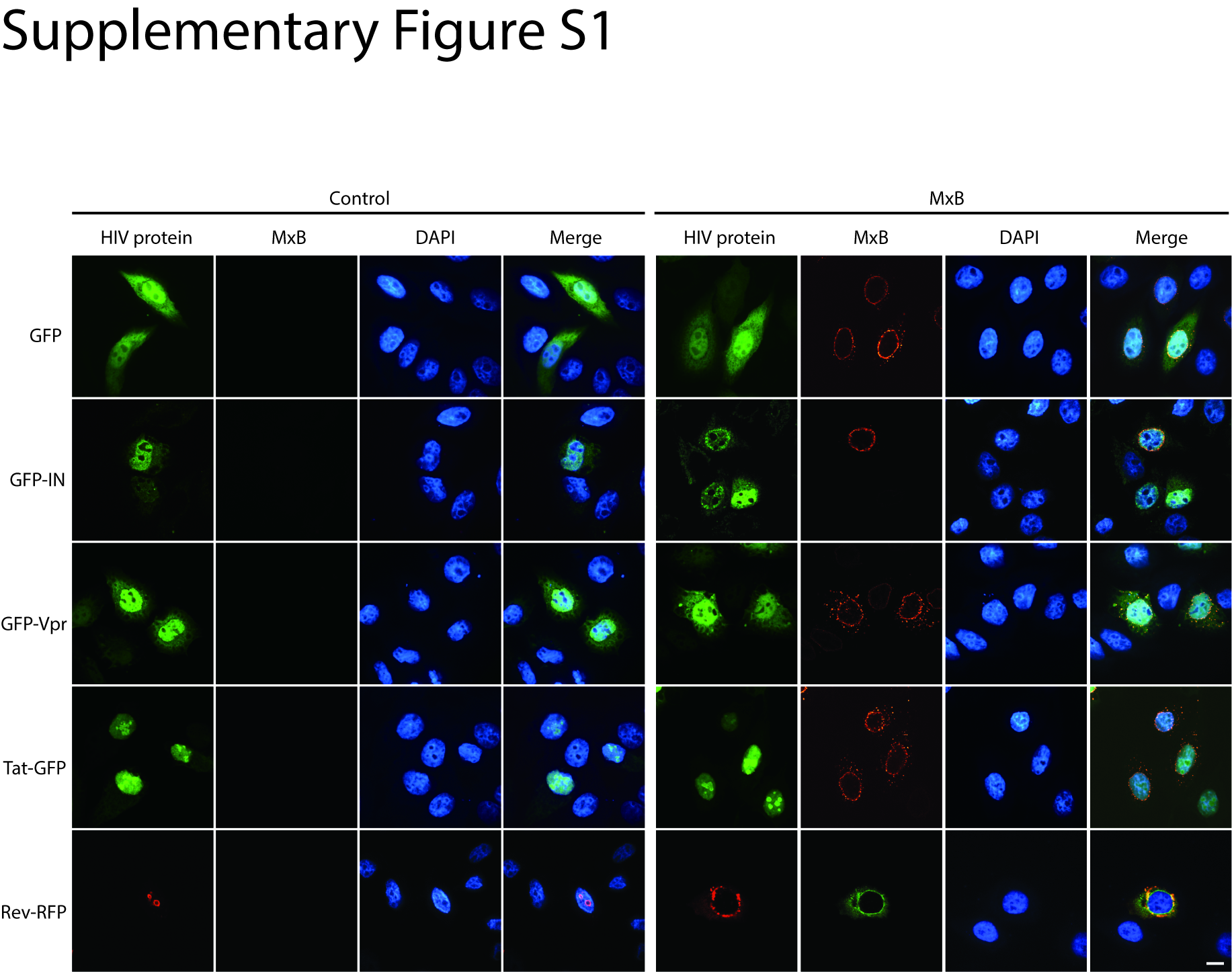

Supplement: Fig_S1.tif [file TEMI_A_1818633_SM7204.tif]
